# Can we use rapid lifetime determination for fast, fluorescence lifetime based, metabolic imaging? Precision and accuracy of double-exponential decay measurements with low total counts

**DOI:** 10.1371/journal.pone.0216894

**Published:** 2019-05-14

**Authors:** Susana Figueiredo Silva, José Paulo Domingues, António Miguel Morgado

**Affiliations:** 1 CIBIT—Coimbra Institute for Biomedical Imaging and Translational Research/INCAS—Institute of Nuclear Sciences Applied to Health, University of Coimbra, Coimbra, Portugal; 2 Department of Physics, Faculty of Sciences and Technology, University of Coimbra, Coimbra, Portugal; Texas A&M University, UNITED STATES

## Abstract

Fluorescence lifetime imaging microscopy (FLIM) can assess cell’s metabolism through the fluorescence of the co-enzymes NADH and FAD, which exhibit a double-exponential decay, with components related to free and protein-bound conditions. *In vivo* real time clinical imaging applications demand fast acquisition. As photodamage limits excitation power, this is best achieved using wide-field techniques, like time-gated FLIM, and algorithms that require few images to calculate the decay parameters. The rapid lifetime determination (RLD) algorithm requires only four images to analyze a double-exponential decay. Using computational simulations, we evaluated the accuracy and precision of RLD when measuring endogenous fluorescence lifetimes and metabolic free to protein-bound ratios, for total counts per pixel (TC) lower than 10^4^. The simulations were based on a time-gated FLIM instrument, accounting for its instrument response function, gain and noise. While the optimal acquisition setting depends on the values being measured, the accuracy of the free to protein-bound ratio α_2_/α_1_ is stable for low gains and gate separations larger than 1000 ps, while its precision is almost constant for gate separations between 1500 and 2500 ps. For the gate separations and free to protein-bound ratios considered, the accuracy error can be as high as 30% and the precision error can reach 60%. Precision errors lower than 10% cannot be obtained. The best performance occurs for low camera gains and gate separations near 1800 ps. When considering the narrow physiological ranges for the free to protein-bound ratio, the precision errors can be confined to an interval between 10% and 20%. RLD is a valid option when for real time FLIM. The simulations and methodology presented here can be applied to any time-gated FLIM instrument and are useful to obtain the accuracy and precision limits for RLD in the demanding conditions of TC lower than 10^4^.

## Introduction

Fluorescence lifetime imaging microscopy (FLIM) can assess cell’s metabolism by using the fluorescence of the metabolic co-enzymes NADH (nicotinamide adenine dinucleotide) and flavins (flavin mononucleotide/flavin adenine dinucleotide—FAD). When properly excited, these molecules exhibit a double-exponential decay, whose intensity profile is given by Eq (1), where *k*_*i*_ is the pre-exponential factor of the decay component with fluorescence lifetime *τ*_*i*_.

$$I\left(t\right)=k_{1}e^{-\frac{t}{\tau_{1}}}+k_{2}e^{-\frac{t}{\tau_{2}}}$$ (1)

The fluorescence decay components of NADH and FAD are related to free and protein-bound conditions [1,2]. For NADH the free condition is responsible for the shorter lifetime component, while for FAD the free condition corresponds to the decay component with longer lifetime. Since the energy production mechanisms of the cells imply the alternation between free and protein-bound states of both co-enzymes, the quantification of these components provides an assessment of the cell’s metabolic state. Although an accurate quantitative assessment of cell’s metabolism through optical techniques requires more sophisticated indicators, as the Optical Metabolic Imaging (OMI) index [3–5], the free to protein-bound ratios of the metabolic co-enzymes have been used in several studies to provide valuable information. Differences in free to protein-bound ratios associated with NADH and FAD fluorescence lifetime were already demonstrated for a wide range of biological samples and proposed as a promising diagnostic method to identify neoplastic diseases [6–8], skin diseases [9] and ocular pathologies [10,11].

When imaging living cells, it is important to acquire the FLIM images as fast as possible to minimize motion artifacts. Fast acquisition is also mandatory in real-time (clinical) imaging applications or for high-throughput screening applications. As the excitation power is limited by photodamage, scanning FLIM approaches are limited in imaging speed. Wide-field imaging techniques, like time-gated FLIM where the fluorescence decay is sampled by a time-gated intensified camera, at several delays after the excitation laser pulse permits fast imaging while using relatively low excitation powers in each pixel [12]. The technique produces an image volume where the set of pixels with the same 2D coordinates correspond to multiple samples of the decay curve describing the fluorescence emission from the conjugated location in the sample. These can be used for computing the decay parameters through non-linear fitting. However, this approach is not suitable for *in vivo* applications with low fluorescence signals as it requires the acquisition of a large number of images and, therefore, many excitation cycles for each gate delay.

The rapid lifetime determination (RLD) method is a set of algorithms that, instead of recording multiple images, uses integrated areas under different regions of the decay curve to determine the decay parameters [13]. A single-exponential decay can be determined using just two images, corresponding to time intervals (gates) set at distinct delays. The parameters of double-exponential decay can be calculated using four images.

Using RLD, the choice of gating parameters, such as gate width (*g*) and time interval between the opening of consecutive gates (*Δt*), affects the signal-to-noise ratio (SNR) of each image and, therefore, the accuracy and precision of the decay parameters. There are several studies on the precision of RLD, mostly focusing just on single-exponential decays [13–16]. They are based on Monte Carlo simulations with Poisson noise, and evaluate several gating schemes including the classical scheme with contiguous, consecutive, gates of equal width, overlapping schemes with constant duration gates and non-adjacent and non-overlapping schemes without imposing gates of equal duration. All studies report on optimal gating parameters that depend on the measured fluorescence lifetime, implying *a priori* knowledge on the fluorescence properties for obtaining the highest precision. Good precisions are possible even at total counts per pixel (TC) as low as 10^4^ with relative standard deviations just 30–40% worse than for least square fitting methods, with the advantage of much faster calculations [13].

Sharman et al [17], were the first to report on double-exponential RLD. They presented analytical expressions for contiguous and 50% overlapping gating schemes, while also using closed form solutions for 25% and 75% overlap. The authors confirmed that double-exponential decays are more demanding and require higher TC values to achieve a given precision. The optimal gating scheme depends not only on the value of the shorter lifetime but also on the ratio between the two lifetimes and on the ratio between fractional contributions to the decay. It was found for TC equal to 10^4^ that, when the lifetimes are known, the relative standard deviation (RSD) in the fractional contributions was less than 5% for contiguous gating and 7% for 50% overlap gating. Much better precisions on the fractional contributions were obtainable at higher TC values since the standard deviation of these parameters is inversely proportional to the square root of TC. When the four decay parameters are unknown, the overlapping RLD has comparable or superior performance to the contiguous RLD methods and shows larger regions of comparable RSD, making it less stringent in terms of measurement conditions. The authors verified that precision degrades as the two lifetimes became closer with reasonable overall precisions requiring a *τ*_*2*_/*τ*_*1*_ value at least 2 and ideally 3 or 4.

More recently, general closed form solutions for double-exponential RLD with arbitrary overlap were presented and used to evaluate the precision of RLD for TC equal to 10^6^ [18]. This study found that the separation between gates, *Δt*, has a greater effect on the precision than the value of gate width *g*.

A common point to all mentioned studies is that *a-priori* knowledge of the expected lifetimes is necessary to define the optimal parameters of acquisition for maximizing the SNR and, consequently, the precision of the measurements with RLD. This is certainly a handicap of the method, particularly when analyzing *in vivo* biological samples using endogenous fluorophores. Although the fluorescent species may depend only on the type of tissue being analyzed, their lifetimes are modulated by their dynamic microenvironment. At best, we can predict the range of fluorescence lifetimes expected, based on the type of sample and on the spectral characteristics of the instrument.

The studies on RLD performance seldom report on accuracy, since the vast majority of them assumes high TC values, where accuracy is not a problem [19]. Heeg [20] reported on the accuracy of the RLD algorithm with gates of equal width and verified that the inaccuracies in the decay parameters were very small compared with the random errors. His analysis was restricted to single-exponential decays. Chang [18] studied the accuracy of the decay parameters for double-exponential decays and reported significant errors when the two fluorescence lifetimes were very similar. These accuracy errors were rare in the regions of the parameter space yielding optimal precision.

It is important to note that all published studies consider the application of RLD using ideal rectangular integration areas. In time-gated FLIM instruments, the output data corresponds to the convolution between the temporal profile of the excitation source, the fluorescence decay profile and the temporal gate profile of the instrument. The convolution between the profiles of the source and the gate corresponds to the instrument response function (IRF) and can be measured experimentally [21]. The IRF of FLIM systems based on gated optical intensifiers departs from the ideal rectangular shape and its width only approximates the nominal width [22]. This deviation results from the intensifier properties and leads to accuracy errors.

Another source of accuracy errors is the finite width of the excitation laser pulse. RLD assumes that the illumination profile on the detector corresponds to the true fluorescence decay of the sample. This is only valid when the laser pulse width is at least two orders of magnitude shorter than the fluorescence lifetime. Otherwise, the convolution between the laser pulse and the fluorescence decay widens the illumination profile and degrades RLD accuracy. Therefore, expensive femtosecond lasers are required when measuring sub-nanosecond fluorescence lifetimes. IRF deconvolution algorithms can be used to improve the accuracy of RLD, as we demonstrated recently for single-exponential decays, opening the possibility of using low cost pulsed diode laser sources to determine accurately fluorescence lifetimes in the sub-nanosecond range [23].

Here we analyze the accuracy and precision of the RLD method for double-exponential decays, considering a time-gated fluorescence lifetime microscope that we setup for metabolic imaging. For this purpose, we performed numerical simulations of endogenous fluorescence decay measurements, accounting for the IRF of the instrument. Noise was modelled according to McGinty et al. [24] considering the measured performance of the intensified camera. As the instrument end goal is to obtain *in vivo* real-time metabolic information from living tissues, we restricted our analysis to double-exponential decays and TC below 10^4^. This last requirement stems from the need of keeping the total acquisition time as low as possible. Real-time imaging precludes the use of frame summing to increase the TC value. Taking in consideration the analog- to-digital (A/D) converter resolution of time-gated cameras, which in the case of our instrument is 12-bits, and the fluorescence lifetimes associated with endogenous fluorescence, it is possible to conclude that the TC will always be lower than 10^4^ even when using the full dynamic range of the camera on the brightest of the four images required by the RLD algorithm. Low TC values can also be imposed by the weak endogenous fluorescence of the metabolic co-enzymes, particularly of FAD that has a lower concentration on the tissues and lower fluorescence cross-section than NADH. This is, for example, the case of cornea endogenous fluorescence as we verified both with one-photon and two-photon excitation FLIM [25,26].

## Methods

### The RLD algorithm for double-exponential decays

Measuring the parameters of the double-exponential decay given by Eq (1), with the RLD algorithm, implies the acquisition of four fluorescence images (*D*_*0*_, *D*_*1*_, *D*_*2*_ and *D*_*3*_), corresponding to gates of equal width *g*, with a constant time interval *Δt* between the beginning of any two consecutive gates. The expressions required to calculate the arrays corresponding to the four decay parameters were obtained by Chang [18]:

$$\tau_{1}=\frac{-\Delta t}{ln\left(y\right)}$$ (2)

$$\tau_{2}=\frac{-\Delta t}{ln\left(x\right)}$$ (3)

$$\alpha_{1}=\frac{k_{1}}{k_{1}+k_{2}}=\frac{-{\left(xD_{0}-D_{1}\right)}^{2}ln\left(y\right)}{\Delta t\left(x^{2}D_{0}-2xD_{1}+D_{2}\right)\left[1-{\left(\frac{xD_{1}-D_{2}}{xD_{0}-D_{1}}\right)}^{\frac{g}{\Delta t}}\right]}$$ (4)

$$\alpha_{2}=\frac{k_{2}}{k_{1}+k_{2}}=\frac{-Rln\left(x\right)}{\Delta t\left(x^{2}D_{0}-2xD_{1}+D_{2}\right)\left(x^{\frac{g}{\Delta t}}-1\right)}$$ (5)

where *x* and *y* are given by

$$x=\frac{\left(-P-\sqrt{DISC}\right)}{2R}$$ (6)

$$y=\frac{\left(-P+\sqrt{DISC}\right)}{2R}$$ (7)

with

$$R=D_{1}D_{2}-D_{2}D_{0}$$ (8)

$$P=-D_{1}D_{2}+D_{3}D_{0}$$ (9)

$$Q=D_{2}D_{2}-D_{3}D_{1}$$ (10)

DISC=PP−4RQ (11)

### Time-gated fluorescence lifetime microscope

The simulations presented are based on the features of a time-gated FLIM instrument that we developed for research on metabolic imaging of living tissues, based on FAD fluorescence imaging. The light source is a pulsed diode laser (LDH-P-C-440M, Picoquant, Berlin, Germany), with a central wavelength of 440 nm and a pulse rate up to 40 MHz, controlled by a multichannel picosecond laser driver (PDL 828 “Sepia II”). The laser peak power is adjustable by the user. This adjustment affects the pulse width that can vary between 63 and 190 ps (FWHM).

Time-gated acquisition is based on a gated-intensified camera (PicoStar HR, LaVision, Goettingen, Germany) composed by a Microchannel Plate (MCP), a High Rate Intensifier (HRI), accepting gates down to 200 ps at a rate between 20 and 80 MHz, a precision delay generator and a 640x480 pixels CCD camera, with 12-bit A/D resolution. A detailed description of the instrument is available in [26].

### Fluorescence decay simulations

Simulations were performed to evaluate the precision and the accuracy of the RLD method when analyzing data acquired with the time-gated FLIM instrument described in the previous section. All simulations were performed using Matlab R2015b (Mathworks, Natick, MA, USA).

A sample representative of endogenous fluorescence was simulated by generating 100x100 data arrays for the four decay parameters, based on data measured on the epithelium of the porcine cornea by two-photon excitation FLIM [25]. The reference values were: *τ*_*1*_ = 550 ± 70 ps; *τ*_*2*_ = 2470 ± 70 ps; *α*_*1*_ = 0.65 ± 0.083; *α*_*2*_ = 0.35 ± 0.083. These lifetimes were attributed to FAD fluorescence since they were measured over a detection band between 500 to 575 nm, above NADH emission band, and because the inherent optical sectioning of two-photon excitation decreased substantially the possibility of detecting fluorescence from stromal collagen. While the values reported for free FAD lifetime are consistent, the values reported for the protein-bound component diverge. While some authors report values around 100 ps [27,28], others present values between 400 ps and 500 ps [29,30].

From the sample arrays and using a time step of 1 ps, we obtained a volume of 25000 data arrays of 100x100 elements, where the element (i,j) of the *m*^*th*^ array is the value of the decay curve corresponding to the decay parameters of the (i,j) coordinates of the sample, for the *m*^*th*^ time step, as given by [21]

$$I\left(m\right)=N\left\{\left[H\left(m\right)+\frac{1}{exp\left(\frac{T}{\tau_{1}}\right)}\right]\alpha_{1}exp\left(-\frac{m}{\tau_{1}}\right)+\left[H\left(m\right)+\frac{1}{exp\left(\frac{T}{\tau_{2}}\right)}\right]\alpha_{2}exp\left(-\frac{m}{\tau_{2}}\right)\right\}$$ (12)

where *H(m)* is the value of the Heavyside function for the *m*^*th*^ time step and *T* is the period of the train of excitation laser pulses. *N* is a parameter used to control the total number of counts on the decay curve.

**Fig 1:**
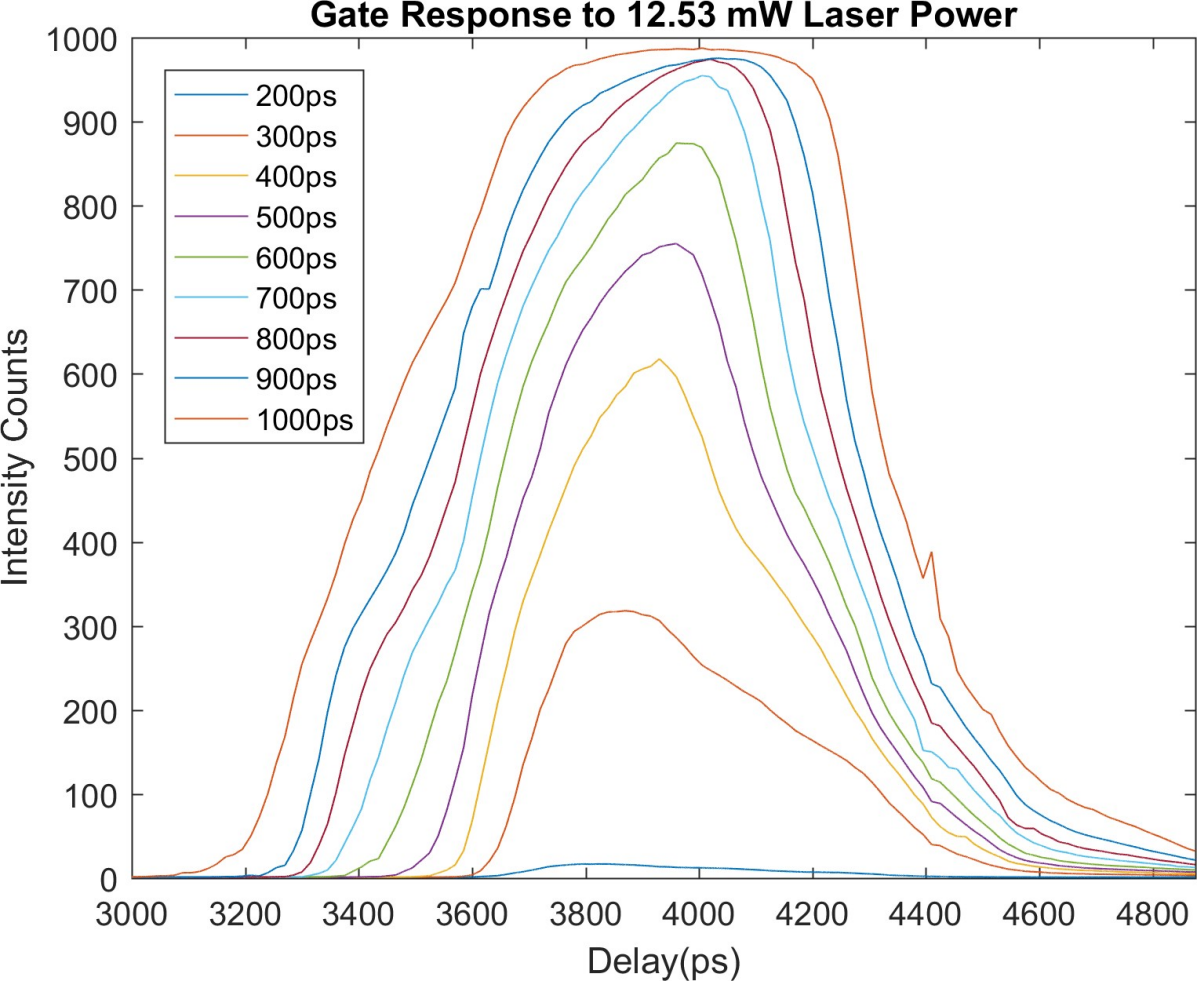
IRF curves for each nominal gate width mode available in the time-gated FLIM instrument, with laser power set to 12.53 mW.

Gating was simulated by convolving the fluorescence decay data with the IRF of the FLIM system. The IRF was measured by directly imaging a white diffusing paper and acquiring a timing scan profile for each nominal gate width. The measurements were performed for several laser power settings, since these settings affect the laser pulse width. As Fig 1 shows, the gate profile shape departs greatly from the ideal rectangular shape.

Noise was added to the data arrays. For the gated intensified camera used in our system, and considering that the photon signal follows Poisson statistics, McGinty et al. showed that noise can be modelled by [24]:

$$\sigma_{I}^{2}=k^{2}EN_{ph}+\sigma_{k}^{2}N_{ph}^{2}+\sigma_{CCD}^{2}$$ (13)

where *k* is a conversion constant, corresponding to the number of digital counts per detected photon (also known as the camera gain), which depends on the image intensifier voltage, *E* is the excess noise factor, *N*_*ph*_ is the number of detected photons, *σ*_*k*_ is the standard deviation associated with fluctuations in the gain *k* and *σ*_*CCD*_ is the noise associated with CCD, like readout noise and dark current noise. On our simulations, we did not consider the noise related to fluctuations in the *k* constant, which is significant only for very high MCP voltages, above the values used in our instrument [24]. Regarding the CCD, we considered just the readout noise (2.28 digital counts). The *k* values were obtained from measurements provided by the manufacturer of the gated intensified camera. The excess noise factor was calculated by interpolating the values presented by McGinty et al. for a similar camera [24]. The values used in the simulations are presented in Table 1.

**Table 1:** Camera parameters used in the simulations.

MCP Voltage (V)	*k* (digital counts /detected photon)	*E*
500	1.05	2.203
550	2.41	1.842
600	5.20	1.653
650	11.1	1.461
700	21.6	1.315
750	45.0	1.158

Conversion constant (gain), *k*, and excess noise, *E*, used in the simulations for different values of MCP voltage.

As the simulations were based in 100x100 data arrays, a total of 10^4^ decay were considered for each simulated condition. The photon count depended on the camera gain. The goal was to simulate the usage of the full CCD dynamic range on the brighter image of the four-image set required by the RLD algorithm, maximizing the number of detected photons and the signal-to-noise ratio. This implies to have always an average pixel value on the first image close to the full-scale range of the camera (4096 counts). The total counts (TC) were calculated by summing the average pixel value of each of the four data arrays used by the RLD algorithm.

## Results

### Validation of simulation algorithm

The simulation algorithm was validated against images from samples with double-exponential fluorescence decay, acquired with the time-gated FLIM instrument. The samples were a mixture of two standard fluorophores in a solution of methanol (MeOH): Erythrosine B and Coumarin 153. Both dyes were purchased from Sigma Aldrich (Darmstadt, Germany). The reagent methanol was of analytical grade (Labbox Labware Barcelona, Spain). Individual solutions of each dye were prepared with concentration of 1 μM and their fluorescence decays, acquired with the Time-Gated microscope, were fitted to a mono-exponential decay using FLIMfit software [21]. Fluorescence lifetime for Erythrosine B in methanol was measured to be 469.24 ± 7.37 ps, while for Coumarin 153 was measured to be 3.94 ± 0.02 ns. These values were confirmed using a Time -Correlated Single Photon Counting lifetime microscope (MicroTime 100, PicoQuant GmbH, Berlin, Germany).

From those solutions, and after dilution in methanol, we prepared a mixture of the two fluorophores. 90% of the mixture volume was from Erythrosine B and 10% was from Coumarin 153. Time-Gated microscope nominal gate width was set to 1000ps and laser to 90% of its power. The gain constant *k* was varied between 1.05 and 45 counts/electron. Measurements were made on 5 solution samples. RLD analysis was performed at each *k* setting. Reference values were obtained using FLIMfit with datasets of 232 images acquired with the time-gated microscope for *k* = 1.05. The time-shift between each image of the datasets was 100 ps. Since Coumarin 153 quantum yield is higher than that of Erythrosine B, the fractional contribution *α*_*1*_ was expected to be lower than 0.9. This was confirmed by the measured reference values: *τ*_*1*_ = 519.32 ps; *τ*_*2*_ = 4118.11 ps; *α*_*1*_ = 0.873; *α*_*2*_ = 0.127. The reference free to protein-bound ratio (*α*_*2*_/*α*_*1*_) is, therefore, equal to 0.145.

**Fig 2:**
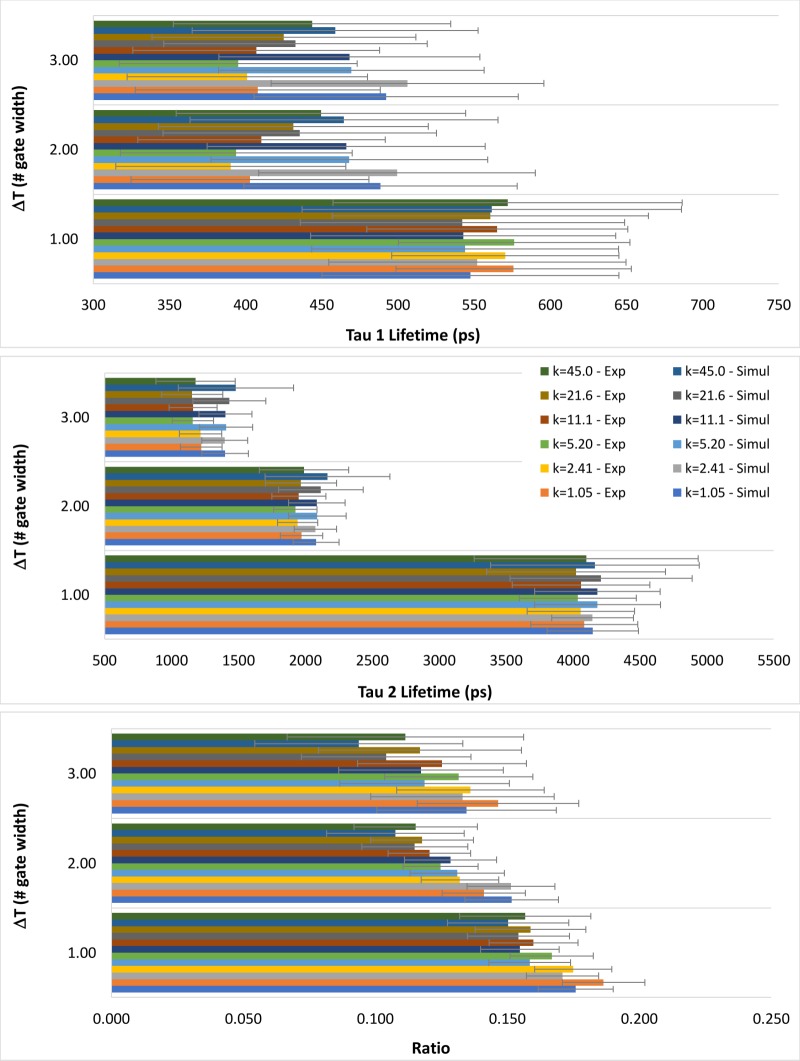
Comparison between the mean values of the decay parameters *τ*_*1*_, *τ*_*2*_ and *α*_*2*_/*α*_*1*_ ratio, obtained from simulated and experimental data using the RLD algorithm. Results are presented for three values of *Δt* (1, 2 and 3 times the gate width). Error bars correspond to ± SD (standard deviation).

For validation purposes, we ran simulations of a fluorophore system with double exponential decay with parameters equal to the reference values obtained from the mixture Erythrosine B and Coumarin 153 in Methanol. Fig 2 presents the comparison between *τ*_*1*_, *τ*_*2*_ and *α*_*2*_/*α*_*1*_ ratio from simulated and experimental data, using the RLD algorithm, for three different values of *Δt* (1, 2 and 3 times the gate width).

The graphs of Fig 2 do not reveal significant differences between simulation and experimental results for corresponding parameters, for any of the three gating schemes tested. This was confirmed by applying the t-test between simulation and experimental samples of a given parameter, for a significance level of 0.05. No statistically significant differences were found.

### Simulation results

Following exploratory simulation trials with TC values higher than 10^6^ and benefiting from several experiments on the time-gated FLIM instrument, we fixed the nominal gate width at 1000 ps and the laser power at 90%. These parameters give the best compromise between higher sensitivity and better accuracy and precision. Therefore, all the results presented here were obtained with these settings.

**Fig 3:**
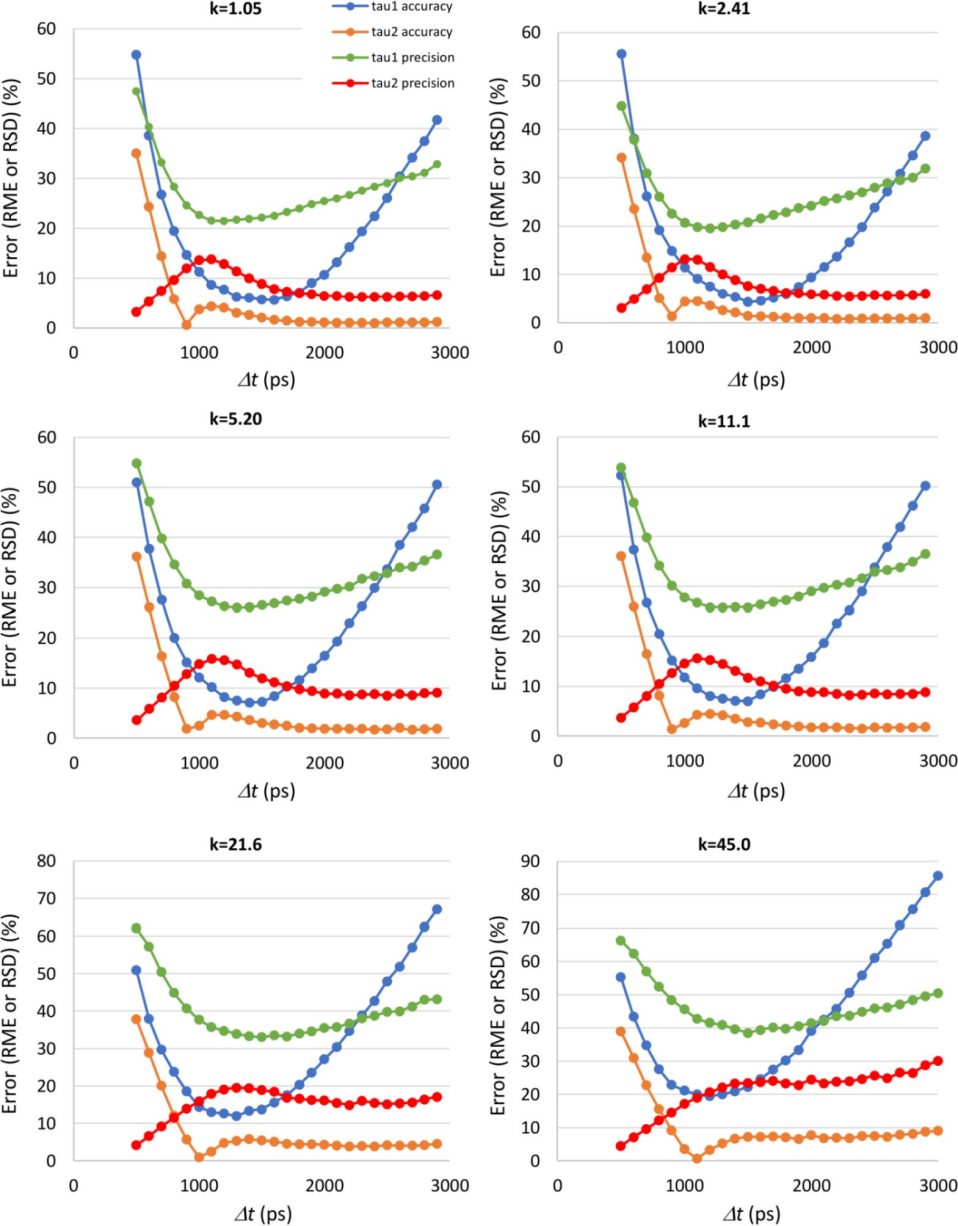
Accuracy and precision of endogenous fluorescence lifetimes obtained by simulation as a function of the gate separation *Δt*, for the different gains of the gated intensified camera (*k*). The accuracy is measured by the relative mean error (RME) and the precision by the relative standard deviation (RSD).

**Fig 4:**
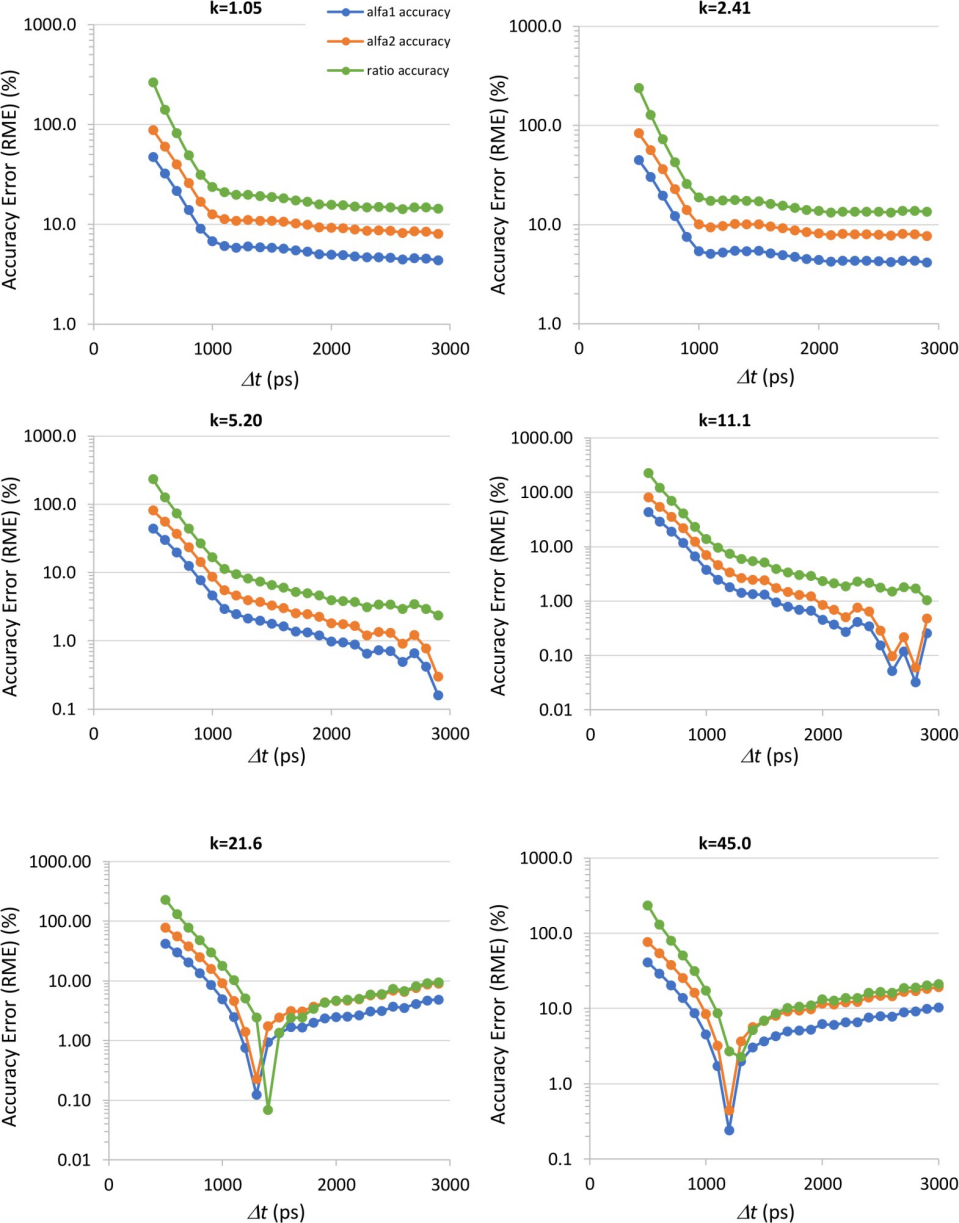
Accuracy of endogenous fluorescence decay parameters *α*_*1*_ and *α*_*2*_ and of the free to protein-bound ratio α_2/_α_1_ obtained by simulation as a function of the gate separation *Δt*, for the different gains of the gated intensified camera (*k*). The accuracy is measured by the relative mean error (RME).

**Fig 5:**
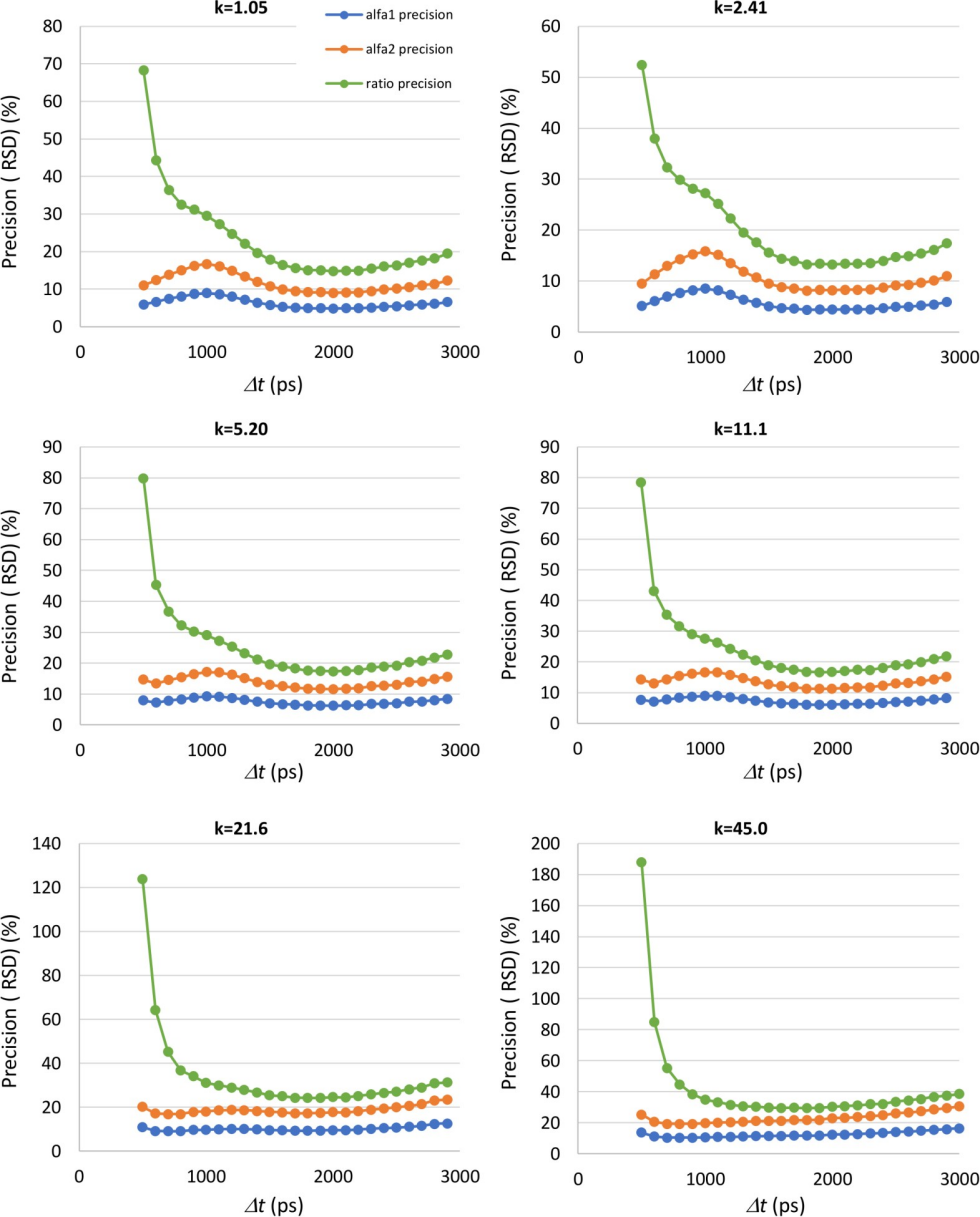
Precision of endogenous fluorescence decay parameters *α*_*1*_ and *α*_*2*_ and of the free to protein-bound ratio *α*_*2*/_*α*_*1*_ obtained by simulation as a function of the gate separation *Δt*, for the different gains of the gated intensified camera (*k*). The precision is measured by the relative standard deviation (RSD).

Using the endogenous fluorescence decay parameters listed in section 2.3, we simulated RLD measurements for the different *k* values of Table 1, to study the influence of gate separation, *Δt*, on the accuracy and precision of the decay parameters. Although the total counts per pixel depend on gate separation, the average counts on the peak image were kept constant and close to the maximum (4094) across all simulations. Fig 3 shows the accuracy and the precision for the fluorescence lifetimes *τ*_*1*_ and *τ*_*2*_. Figs 4 and 5 present the accuracy and the precision for the fractional contribution *α*_*1*_ and *α*_*2*_ and for the free to protein-bound ratio *α*_*2*_/*α*_*1*_. The accuracy is given by the relative mean error (RME) and the precision by the relative standard deviation (RSD), defined according to

$$RME=\frac{\left|c_{meas}-c_{ref}\right|}{c_{ref}}\text{x}100\%$$ (14)

$$RSD=\frac{\sigma_{meas}}{c_{meas}}x100\%$$ (15)

where *c*_*meas*_ is the average measured value of a given decay parameter, *c*_*ref*_ is the reference value for that decay parameter and *σ*_*meas*_ is the standard deviation associated to the measure of the decay parameter.

On the accuracy of the fluorescence lifetimes (Fig 3), we can conclude that it is always better for the longer lifetime *τ*_*2*_ than for *τ*_*1*_. This is an expected result since *τ*_*1*_ is in the order of 500 ps making its measurement highly affected by the IRF of the time-gated FLIM instrument. The accuracy of *τ*_*2*_ is always better than 10% for gate separations higher than 800 ps, with a pronounced minimum for values *Δt* in the range from 900 to 1100 ps. It is important to note that the gate separation that yields this minimum accuracy error depends highly on the decay parameters. So, tuning the gating scheme for maximum accuracy is only possible with *a priori* knowledge on the decay parameters. The accuracy of *τ*_*1*_ is clearly better for lower gains (*k* ≤11.1). The pattern of variation is similar throughout all camera gains, with an optimal region for gate separations between 1000 and 2000 ps.

The fluorescence lifetime precision also exhibits a variation pattern that is almost independent from the camera gain. Once again, the performance is better for *τ*_*2*_ than for *τ*_*1*_, a result that can also be explained by the effect of the IRF width. The precision of *τ*_*1*_ is clearly better for low gains (*k* ≤ 2.41). There is an optimal region for gate separations between 1000 and 1800 ps, but the RME is only lower than 20% for *k* = 2.41.

The precision of *τ*_*2*_ is less affected by the gate separation *Δt*. For the lower gains (*k* ≤ 11.1), it is always better than 10% for gate separations equal or higher than 1800 ps. The precision error also decreases for low values of gate separation (< 700ps). However, the performance for the other parameters in this region of *Δt* values is clearly inadequate.

The accuracy of the fractional contributions *α*_*1*_ and *α*_*2*_ and of the *α*_*2*_/*α*_*1*_ ratio is presented in Fig 4. For gains *k* ≥ 5.2 it is possible to identify clear minimums for the accuracy error. The gate separation for which these minimums occur depends on the gain setting. It also depends on the nominal values of the pre-exponential parameters as we will show. From the point of view of a real application, where *a-priori* knowledge on *α*_*1*_ and *α*_*2*_ is not available, it is desirable to operate in a settings range where performance is as stable as possible. For *α*_*1*_, *α*_*2*_ and ratio accuracies this is obtained at *k* ≤ 2.41 and *Δt* > 1000 ps. In this operating region, it is possible to obtain an accuracy error for the free to protein-bound ratio around 15%.

The precision of *α*_*1*_, *α*_*2*_ and of the free to protein-bound ratio almost stabilizes for gate separations between 1500 and 2500 ps. This happens for all *k* values. In this range of gate separations, the precision of the free to protein-bound ratio is near the limit imposed by error propagation. In fact, the ratio is not measured directly but calculated from *α*_*1*_ and α_2._ Knowing the variances associated with the pre-exponential factors, it is possible to obtain the precision limit for the ratio *r*. If we consider the definition of the *α*_*2*_*/α*_*1*_ ratio and knowing that, in the absence of noise, the correlation coefficient, *ρ*, between *α*_*1*_ and *α*_*2*_ is equal to -1, we obtain

$$\sigma_{r}^{2}\approx {\left(\frac{\partial r}{\partial \alpha_{1}}\right)}^{2}\sigma_{\alpha_{1}}^{2}+{\left(\frac{\partial r}{\partial \alpha_{2}}\right)}^{2}\sigma_{\alpha_{2}}^{2}+2\frac{\partial r}{\partial \alpha_{1}}\frac{\partial r}{\partial \alpha_{2}}\rho \sigma_{\alpha_{1}}\sigma_{\alpha_{2}}\approx \frac{1}{\alpha_{1}^{2}}\left[r^{2}\sigma_{\alpha_{1}}^{2}+\sigma_{\alpha_{2}}^{2}+2r\sigma_{\alpha_{1}}\sigma_{\alpha_{2}}\right]$$ (16)

where $\sigma_{r}^{2}$, $\sigma_{\alpha_{1}}^{2}$ and $\sigma_{\alpha_{2}}^{2}$are the variances associated with *r*, *α*_*1*_ and *α*_*2*_, respectively. This equation shows that higher values of the ratio will inherently have lower precision.

**Fig 6:**
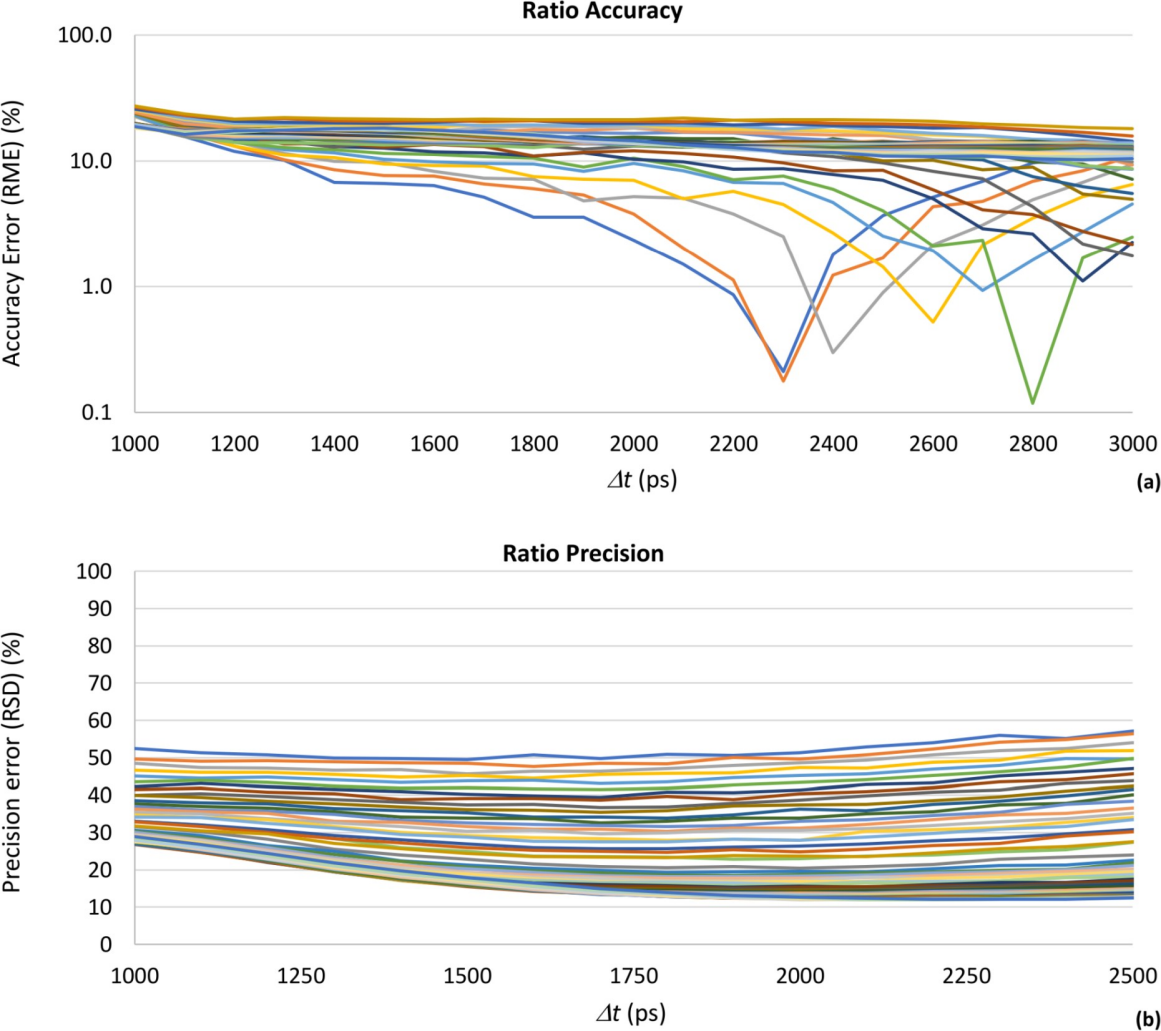
Accuracy (a) and precision (b) of the free to protein-bound ratio α_2_/α_1_ for *k* = 2.41; *Δt* = 1800 ps, as a function of gate separation *Δt*, for a free to protein-bound ratio range between 0.25 and 2.846. On the precision graph, the ratio decreases from top to bottom. On the accuracy graph, the sequence of ratio curves is not regular.

We performed additional simulations to evaluate the accuracy and precision of the RLD algorithm for different values of free to protein-bound ratio. These simulations were done for *k* = 2.41 since this was the gain value that offered better overall performance on the previous simulations. We varied the *α*_*2*_ factor from 0.2 to 0.74 in 0.01 steps, corresponding to a ratio range from 0.25 to 2.846, and varied the gate separation to cover the ranges of stable accuracy and precision identified on the previous simulations. The results are presented in Fig 6A and 6B

Fig 6A and 6B show clearly that the accuracy and the precision of the free to protein-bound ratio vary with its mean value, as expected from the previous simulations. These results demonstrate that, without having information on the expected values of the α_2_/α_1_ ratio, it is not possible to find an optimal acquisition setting, since the accuracy and precision of the measurement of the metabolic parameter depend on its nominal value. On the contrary, if we know at least the expected range of the free to protein-bound ratio, then the simulations make allow to assess the performance of the different gating schemes and to determine the best acquisition parameters for any total counts, gate widths and gate separations. The precision curves confirm that higher values of free to protein-bound ratio will have worse precision due to the limits imposed by error propagation from the measurements of *α*_*1*_ and *α*_*2*_. Likewise, the figures confirm that no value of gate separation offers measurement performance independent of the ratio value being measured. They also show that, for any ratio value, its precision is quite stable for gate separations between 1500 and 2500 ps.

With this set of simulations, it was possible to verify that the accuracy and the precision associated to the value of the free to protein-bound ratio obtained by RLD algorithm not only depend on the adopted gating scheme, but also on the nominal value of the ratio. For the ranges of gate separation and *α*_*2*_*/α*_*1*_ ratio considered, the accuracy error can be as high as 30% and the precision error can reach a maximum value around 60%. Precision errors lower than 10% cannot be obtained.

**Fig 7:**
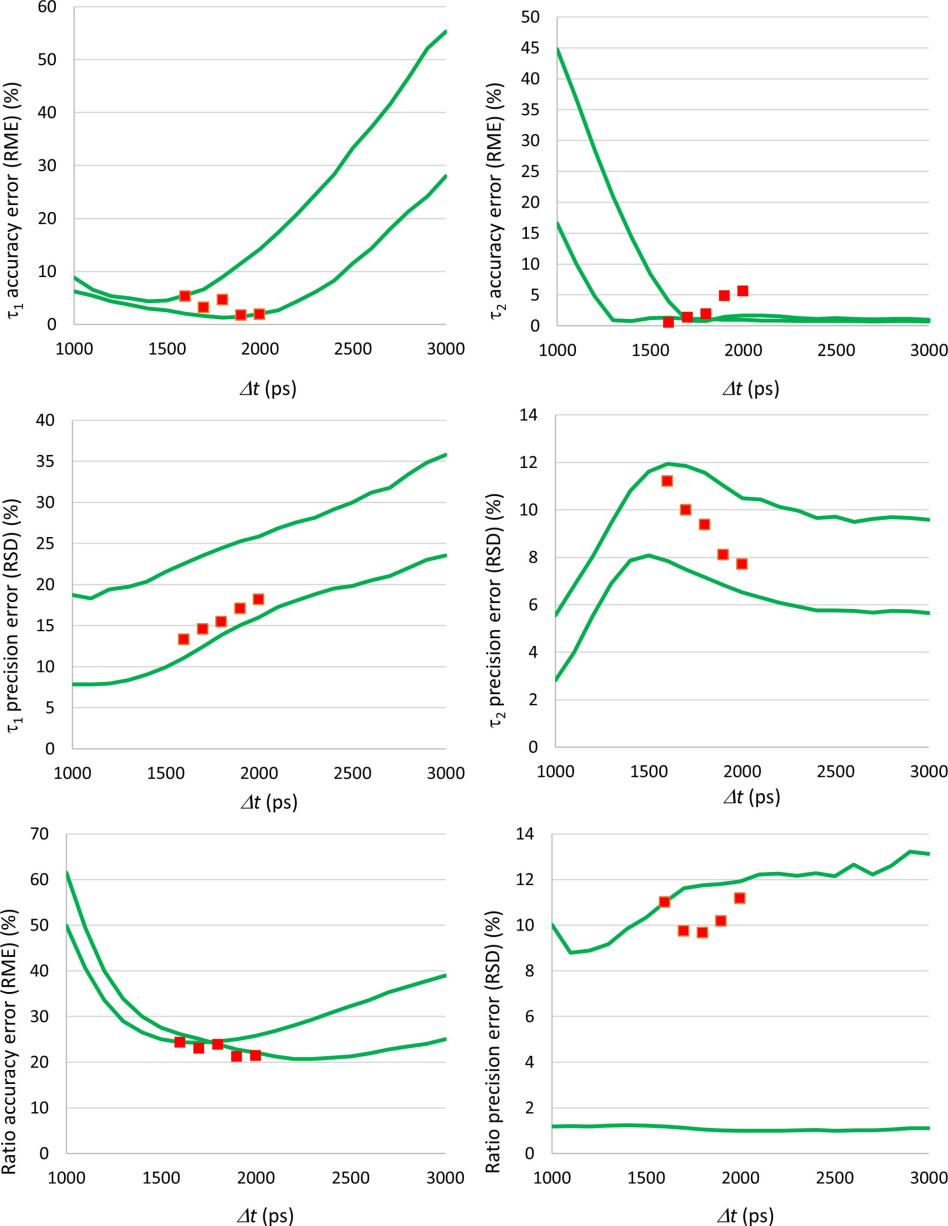
Evaluation of the best gating scheme for a solution sample. Simulations used as expected lifetimes τ_1_ = 500 ps and τ_2_ = 4000 ps. The α_1_ value was varied from 0.7 to 0.9. The continuous lines correspond to the accuracy and precision ranges obtained for the decay parameters. The dots indicate the accuracy and the precision errors measured experimentally for the interval of gate separations that were considered to provide the best overall measurement performance (1600 to 2000 ps).

Here we present an example of how the simulations could be used to help defining the best gating scheme for a double-decay fluorescent sample. We took an unused sample from the solution previously employed in the validation process. In this case, we knew exactly the reference decay parameters: *τ*_*1*_ = 519.32 ps; *τ*_*2*_ = 4118.11 ps; *α*_*1*_ = 0.873; *α*_*2*_ = 0.127. However, for simulation purposes we considered that we had only an idea of the expected lifetimes and of the range of α_2_/α_1_ ratios. So, we ran simulations considering *τ*_*1*_ = 500 ps, *τ*_*2*_ = 4000 ps, and *α*_*1*_ ranging from 0.7 to 0.9. By examining the simulation results, we considered that the best overall performance was obtained for gate separations between 1600 ps and 2000 ps. Therefore, we selected these gate separations to measure the decay parameters of the sample using RLD. As Fig 7 shows, the accuracy and the precision of the measurements was in the range predicted by the simulations.

## Discussion

We evaluated the performance of the RLD algorithm on the demanding regime of TC lower than 10^4^, by carrying out several simulations of endogenous fluorescence decay. All simulations were carried out for a nominal gate width of 1000 ps, a selection based on our experience with the time-gated FLIM instrument and on simulations with high total counts per pixel. We confirmed the literature reports stating that the gate separation has a much higher impact of the precision of RLD than the gate width [18]. We ran simulations using ideal rectangular gates, varying the gate width from 500 ps to 2500 ps, while keeping a fixed gate separation of 1800 ps. The average counts per pixel (TC) were lower than 10^4^. The precision of the decay parameters did not vary more than 2.3% for τ_1_ and less than 0.5% for the other decay parameters. The accuracy varied a maximum of 4.7% for τ_1_ and less than 2.8% for the other parameters. These results validate our decision of using a constant gate width.

As expected, the gate separation that yields the maximum fluorescence lifetime accuracy depends on the values of the decay parameters. Our results show that the measurement accuracy is always better for the longer lifetime, *τ*_*2*_, a result that reflects the influence of the system IRF, which is not considered when applying the RLD algorithm. The shorter lifetime τ_1_ is in the order of 500 ps, a value that is less than one order of magnitude shorter than the IRF width of the simulated instrument, since for the 90% power setting of the excitation laser used in the simulations, the laser pulse alone has a width larger than 100 ps. Improving the accuracy on the value of the shorter wavelength would require the use of expensive femtosecond lasers or to modify the RLD algorithm to account and correct the effect of the IRF width. We concluded that the accuracy of τ_2_ is always better than 10% for gate separations higher than 800 ps and that the accuracy of *τ*_*1*_ is clearly better for gains equal or lower than 11.1, with an optimal region for gate separations between 1000 and 2000 ps.

The effect of the instrument’s IRF in the measurement accuracy can be corrected by deconvoluting the IRF from the acquired data. We already demonstrated the benefits of this approach on accuracy improvement for single-exponential decays [23]. The procedure is based in the iterative convolution of the measured IRF with synthetic fluorescence decay data, generated in a similar way to what we described in the “Fluorescence decay simulations” section. However, while for single-exponential decays, the RLD deconvolution is relatively straightforward, since the decay equation is easily linearized and the lifetime deviation always occurs in the direction of higher lifetimes, the same does not apply to double-exponential decays, where the deconvolution problem becomes an ill-poised problem. As far as we know, no RLD deconvolution method for double-exponential decays was ever reported. Currently we are evaluating analytical methods using a polynomial approximation to the measured IRF.

Concerning precision, the performance for *τ*_*1*_ is clearly better for low gains (*k* ≤ 2.41), with an optimal region for gate separations between 1000 and 1800 ps. However, the precision error is almost always larger than 20%. The precision of the longer lifetime is better, with RSD values better than 10% for gate separations equal or higher than 1800 ps at low gains (*k* ≤ 11.1).

Our simulations revealed that the accuracy of the fractional contributions *α*_*1*_ and *α*_*2*_ and of the *α*_*2*_/*α*_*1*_ ratio remains quite stable for *k* ≤ 2.41 and *Δt* > 1000 ps. This is an important result since *a priori* knowledge on *α*_*1*_ and *α*_*2*_ is not easy to obtain as the values of these parameters depend highly on the cell’s metabolic state. Therefore, it is advisable to operate the instrument with settings where the measurement performance is as independent as possible from the adopted gating scheme. For the mentioned operating region, it is possible to obtain a ratio accuracy error around 15%. The precision of *α*_*1*_, *α*_*2*_ and of the free to protein-bound ratio is almost constant for gate separations between 1500 and 2500 ps, for all *k* values, with the precision of the ratio near the limit posed by error propagation. The best precisions are obtained for *k* = 2.41 but the ratio precision is always worse than 10%. The optimal operating regions in terms of precision and accuracy are compatible, allowing the adoption of operating parameters that maximize both accuracy and precision.

Finally, we studied the RLD performance for a range of values of the free to protein-bound ratio, while varying the gate separation to cover the ranges of stable accuracy and precision previously identified. The results show that is not possible to find an optimal acquisition setting for the time-gated fluorescence lifetime microscope when using the RLD algorithm, without having previous information on the decay parameters. This happens because the quality of the measurement of the *α*_*2*_*/α*_*1*_ ratio depends on its nominal value: higher values of the ratio will have worse precision and better accuracy. On the contrary, if knowledge on the expected value or range of values of the *α*_*2*_*/α*_*1*_ ratio is available, the simulations allow to identify the optimal gate separation for any gate width, TC value or system IRF, being useful for setup the measurement for the best possible performance.

The results also show that no value of gate separation offers measurement performance independent of the ratio value being measured. However, for any given ratio value, its precision is quite stable for gate separations between 1500 and 2500 ps. For the ranges of gate separation and free to protein-bound ratio we considered, the accuracy error can be as high as 30% and the precision error can reach a value around 60%. Precision errors lower than 10% cannot be obtained. The best performance is obtained when the intensified camera is operated with the lower gains (≤ 2.41) and image acquisition is setup for gate separations close to 1800 ps. We cannot compare these results with other studies since, as far as we know, there are no published reports on the accuracy and precision of RLD for TC lower 10^4^ and accounting for the noise and IRF of the instrument.

This raises the question of whether metabolic imaging through FLIM should rely on the RLD algorithm. The answer depends highly on the application, namely on the expected range of free to protein-bound ratio values. The physiological range for the free to protein-bound ratio of endogenous fluorophores is relatively narrow, as Skala et al. found for FAD between normal, low grade and high grade precancerous cells in the hamster cheek pouch [28]. Batista et al. reported on the variation of the free to protein-bound ratio of FAD along the porcine corneal epithelium depth. The average value of the ratio varied between 0.67 at a 5 μm depth and 0.52 at a depth of 75 μm. For this ratio range, it is possible to obtain precision errors between 13.1% and 14.1% for TC values lower than 10^4^, which are acceptable for many applications.

RLD should be always an option to consider when doing real-time imaging, particularly in clinical applications, since it allows fast image acquisition. If high precision is mandatory, the only solution is to increase the total number of counts either by summing frames or using pixel binning. Sharman et al. showed that the standard deviation in both *α*_1_ and *α*_2_ is inversely proportional to the square root of the total counts [17]. Although not addressed in this paper, we also ran simulations for TC between 10^4^ and 10^5^. In this condition, RLD performance is quite improved, confirming the results of other authors [16–18], being possible to obtain accuracy errors lower than 5.0% on the free to protein-bound ratio, if we restrict the gain *k* to values equal or lower than 5.2. Moreover, the ratio precision is not degraded by the measurement, being close to the precision defined originally in the sample. The accuracy on the fluorescence lifetimes is also adequate, with errors always below 4%, with the only significant precision degradation occurring for the shortest lifetime. So, for TC higher than 10^4^, RLD is quite advantageous for real-time imaging. However, it can be difficult to obtain those TC values within an acquisition time compatible with the requirements posed by *in vivo* real-time metabolic imaging, particularly when imaging FAD fluorescence. Although one-photon excitation of FAD is safer than UV excitation of NADH, FAD has lower fluorescence quantum yield and its concentration in cells is usually two orders of magnitude lower than the concentration of NADH. It is very difficult to obtain a FAD signal large enough to use the full dynamic range of the CCD cameras installed in time-gated FLIM instruments, which is typically 12 bits. Anyway, even if we managed to obtain a FAD signal around 4000 counts in the brightest image, the TC value would still be lower than 10^4^ unless we chose a gating scheme with considerable overlapping between consecutive gates. For a gate width of 1000 ps, this would imply a gate separation not higher than 300 ps, a condition that results in insufficient measurement performance.

One way of obtaining higher TC values is to repeat the acquisition process and sum the frames acquired for equivalent gates. This approach has the inconvenient of increasing the total acquisition time, which can be unacceptable in clinical applications. According to our assessments, if we use the full dynamic range on the image corresponding to the first gate, obtaining TC = 5 x 10^4^ when using a gate separation of 1000 ps requires summing 8 frames per gate, increasing the acquisition time by the same factor. We need to sum 15 frames for obtaining a total count of 10^5^, while TC = 10^6^ implies summing 147 frames. The number of frames to be summed is higher for larger gate separations and for samples with very week emission, where it is not possible to use the full dynamic range of the camera.

Binning can be an effective solution to increase the TC and, consequently the accuracy and precision of RLD. The drawback is that the spatial sampling is affected, decreasing the maximum observable spatial frequency.

Even though we only simulated samples using lifetimes attributed to FAD, measured on the epithelium of intact porcine corneas [25], the simulation-based methodology presented here can be applied to different samples with different lifetime ranges. In RLD-based time-gated FLIM, the selection of an adequate gating scheme is not trivial, even when there is some *a priori* knowledge on the decay lifetimes. Optimizing the RLD method for one decay lifetime will always degrade the precision and accuracy on the other lifetime, particularly for low counts per pixel conditions. For high counts (10^6^), Sharman et al. [17] showed that is possible to obtain reasonable overall precisions for both lifetimes if the longer lifetime is at least twice the shorter lifetime (3–4 times would be best), providing that α_1_ is larger than α_2_. Little additional precision was gained if a larger τ_2_/τ_1_ ratio was considered. The most challenging conditions occurred when the two lifetimes have close values and when the fractional contribution of the shorter lifetime was much lower than that of the longer lifetime. Using simulations, we get an overall assessment of the accuracy and precision of all decay parameters, for any TC value and gating parameters. This helps to identify the best acquisition settings, which may correspond to an operating region where precision and accuracy are stable and less sensitive to the choice of gating parameters or to small variations in the decay values, instead of optimizing the acquisition for a particular decay parameter.

## Conclusions

We found that the accuracy of the free to protein-bound ratio is stable for low camera gains and gate separations larger than 1000 ps, while its precision is almost constant for gate separations between 1500 and 2500 ps. For the ranges of gate separation and *α*_*2*_*/α*_*1*_ ratio considered, the accuracy error can be as high as 30% and the precision error can reach 60%. Precision errors lower than 10% cannot be obtained. The best performance occurs for low camera gains and gate separations near 1800 ps. When considering the reported physiological ranges of the *α*_*2*_*/α*_*1*_ ratio, the precision errors can be confined to an interval between 10% and 20%.

The simulations presented here were based on a time-gated FLIM instrument setup by us. However, these simulations and the methodology we followed can be used with any time-gated FLIM system based on gated intensified CCD cameras and are useful to obtain the accuracy and precision limits for double-exponential RLD in the demanding conditions of TC lower than 10^4^. The use of picosecond pulsed lasers decreases substantially the cost of the time-gated FLIM instrument but implies measuring the IRF for an accurate simulation of RLD performance.

## Supporting information

S1 File Excel file with data and graphs for Figs 3, 4 and 5. (XLSX)

S2 File Excel file with data and graphs for Fig 6. (XLSX)

S3 File Excel file with data and graphs for Fig 7. (XLSX)
